# Large-scale dynamics have greater role than thermodynamics in driving precipitation extremes over India

**DOI:** 10.1007/s00382-020-05410-3

**Published:** 2020-08-03

**Authors:** Naveen Sudharsan, Subhankar Karmakar, Hayley J. Fowler, Vittal Hari

**Affiliations:** 1 Environmental Science and Engineering Department, Indian Institute of Technology Bombay, Mumbai, 400076 India; 2 Interdisciplinary Program in Climate Studies, Indian Institute of Technology Bombay, Mumbai, 400076 India; 3 Centre for Urban Science and Engineering, Indian Institute of Technology Bombay, Mumbai, 400076 India; 4 School of Engineering, Newcastle University, Newcastle-upon-Tyne, UK; 5 Department of Computational Hydrosystems, Helmholtz Centre for Environmental Research, UFZ, Leipzig, Germany

## Abstract

The changing characteristics of precipitation extremes under global warming have recently received tremendous attention, yet the mechanisms are still insufficiently understood. The present study attempts to understand these processes over India by separating the ‘dynamic’ and ‘thermodynamic’ components of precipitation extremes using a suite of observed and reanalysis datasets. The former is mainly due to changes in atmospheric motion, while the latter is driven mainly by the changes associated with atmospheric moisture content. Limited studies have attributed dynamic and thermodynamic contributions to precipitation extremes, and their primary focus has been on the horizontal atmospheric motion component of the water budget. Our study, on the other hand, implements the decomposition of vertical atmospheric motion, based on the framework proposed by Oueslati et al. (Sci Rep 9: 2859, 2019), which has often been overlooked, especially for India. With the focus on two major and recent extreme events in the Kerala and Uttarakhand regions of India, we show that the vertical atmospheric motion has a more significant contribution to the events than the horizontal atmospheric motion. Further, decomposition of the vertical atmospheric motion shows that the dynamic component overwhelms the thermodynamic component’s contribution to these extreme events, which is found to be negligible. Using a threshold method to define extreme rainfall, we further extended our work to all India, and the results were consistent with those of the two considered events. Finally, we evaluate the contributions from the recently made available CMIP6 climate models, and the results are interestingly in alignment with the observations. The outcomes of this study will play a critical role in the proper prediction of rainfall extremes, whose value to climate adaptation can hardly be overemphasised.

**Electronic supplementary material:**

The online version of this article (10.1007/s00382-020-05410-3) contains supplementary material, which is available to authorized users.

## Introduction

Human-induced climate change is evident, and currently poses a great concern to society, primarily due to its potential to intensify extreme precipitation events around the globe (Min et al. 2011; Pall et al. 2011). Both, observations (Allan et al. 2010; Min et al. 2011; Deser et al. 2012) and simulations from global climate models (Meehl et al. 2000; Kharin et al. 2007) depict increased rainfall extreme events due to a warming climate, but with substantial internal variability in some regions (Fischer and Knutti 2014). In addition, rainfall extremes will increase, even in regions where the intensity of mean precipitation is showing a decreasing trend (Field et al. 2012), which may obviously lead to tremendous ecological and socioeconomic losses. Given these observations, a comprehensive understanding of the space–time variability of rainfall extremes is critical for sustainable water resource and disaster management at the required spatial scales (Gusain et al. 2019).

While explaining the cause, Trenberth (1999) claimed that the intensification of rainfall extremes in a warming world is primarily caused by enhanced availability of atmospheric moisture content. Following this, numerical model-based studies (Allen and Ingram 2002; Kharin et al. 2007) reported that the water vapor content would increase at a rate of 7% for every degree Celsius increase in surface warming. Further, precipitation extremes are expected to increase either at the same rate as the water vapor content or at an even higher rate, if the updraft strength associated with the extreme event increases with warming. This relation closely follows the Clausius–Clapeyron (CC) equation (Allen and Ingram 2002; Held and Soden 2006; Pall et al. 2007) and is referred to as the thermodynamic response of precipitation extremes to warming. However, using observations, studies by Groisman et al. (2005) and Alexander et al. (2006) reported that the increasing trend of precipitation extremes is not spatially uniform around the globe. This discrepancy between CC scaling and observed trend is more evident particularly in tropical countries. This suggests that the CC scaling can be affected by several factors, among which the duration and type of precipitation extremes (Molnar et al. 2015), range of temperatures (Westra et al. 2014), geographical locations (Wasko et al. 2016), and seasonality (Berg et al. 2009) are the considered to be an important factor. This highlights the fact that, along with thermodynamic components, the dynamic elements (mostly dependent on the atmospheric circulation) also have a strong influence on precipitation extremes in many regions (Trenberth et al. 2015; Shepherd 2016; Pfahl et al. 2017).

This discrepancy with the theory in the tropical region was justified by O’Gorman and Schneider (2009a, b) with a large ensemble of climate models. They highlighted that simulations of precipitation extremes in the tropics are not reliable and there exists a considerable uncertainty in representing changes to the extremes due to warming over a range of climate models. Addressing these inconsistencies with a physical basis, they show that improving the simulation of *upward velocities* in a climate model is essential for improving predictions of precipitation extremes, especially for the tropics. They found that one of the critical proxies to estimate the dynamic response of precipitation extremes is the *upward velocities.* In addition, Emori and Brown (2005) pointed out that dynamic components plays a significant role over tropics; and its influence should not be neglected, especially while analyzing the causes for change in precipitation extremes. In continuation, Dairaku and Emori (2006) showed that the enhanced response of precipitation extremes over South Asia arose from dynamic changes rather than thermodynamic changes. Recent studies by Vittal et al. (2016), Ali and Mishra (2018) and Gusain et al. (2019) also reported the importance of dynamic components to precipitation extremes. Thus, these studies highlighted that the responses of dynamic contributions must be considered alongside thermodynamic contributions to understand the changes in precipitation extremes to warming in the tropical regions.

Now we emphasize literature on the causation of Indian summer monsoon rainfall (ISMR), considering the background information on tropical regions, mainly focusing on its extremes. ISMR, being an inherent part of tropical rainfall that happens during June–September (JJAS), plays a significant role in agricultural productivity and thus contributes to the gross domestic product (GDP) of India (Gadgil and Kumar 2006). Therefore, understanding the cause of the recent changes to ISMR and its extremes has massive implications for the country’s water resource planning and policy formation (Mall et al. 2006; Archer et al. 2010); and also for disaster management of the nation (Vittal et al. 2020; Sharma et al. 2020). Here, contrasting findings between studies have attributed recent changes in the characteristics of extreme rainfall to global warming (Rajeevan et al. 2008; Mani et al. 2009) or to local factors, including urbanization (Kishtawal et al. 2010; Vittal et al. 2013). Although it is clear from the literature that rainfall extremes over India have been increasing recently (Goswami et al. 2006; Dash et al. 2009; Singh et al. 2014), the underlying mechanisms responsible are unclear. A recent study by Vittal et al. (2016) provided crucial insight into these aspects by analyzing the sensitivity of rainfall extremes to warming, with respect to both surface air temperature and sea surface temperature. Interestingly, they found negative scaling rates (negative relationship) for most of India, although this relationship becomes positive in most areas when including a moisture component through dew point temperature (Ali et al. 2018; Lenderink and Fowler 2017). However, the dynamic component has not been considered. Moreover, Gusain et al. (2019) showed that the performance of statistical downscaling of precipitation extremes from climate models increases with the inclusion of a dynamic component, ‘*upward velocity*’*,* as one of the predictors.

The present study focuses on understanding the cause of changes in ISMR precipitation extremes by separating the thermodynamic and dynamic contributions. We implement the decomposition of vertical atmospheric motion, based on the framework proposed by Oueslati et al. (2019) using two extreme events which happened recently over Kerala (2018) and Uttarakhand (2013). The former triggered massive flooding in the state and led to the loss of over 400 lives, causing damages amounting to US$ 4.4 billion (Vinod Chandra Menon et al. 2019). Numerous landslides and extensive flooding in the state due to the latter cost 4000 lives (Kala 2014) and produced economic damages of over US$ 3.8 billion (The World Bank 2013). We then extend this analysis for all India. Finally, we compare the dynamic and thermodynamic contributions as obtained from a recently released suite of Coupled Model Intercomparison Project—Phase 6 (CMIP6) climate model outputs with those for the observations to assess the reliability of projections of precipitation extremes.

## Data and methods

The global daily precipitation dataset for 1979—present is obtained from the NOAA Climate Prediction Center-based (CPC) gauge-based analysis of daily precipitation. Here, we preferred to use the dataset from CPC rather than any other observed dataset due to its temporal availability at the daily scale. National Oceanic and Atmospheric Administration (NOAA) Climate Prediction Centre (CPC) created this dataset to provide a set of unified precipitation data with consistent quantity and improved quality. It consists of gauge reports from 30,000 stations across the world, including reports from the World Meteorological Organization Global Telecommunication System (GTS), Cooperative Observer Network (COOP), and other National Meteorological Agencies (Xie et al. 2010; Sun et al. 2018).

To understand the large-scale features along with the moisture budget during the extreme events over India, we use two different reanalysis products: the Japanese 55-Years Reanalysis (JRA-55) dataset (Ebita et al. 2011; Kobayashi et al. 2015) provided by the Japan Meteorological Agency (JMA) and the European Centre for Medium-Range Weather Forecasts (ECMWF) Interim Reanalysis (ERA-Interim) dataset (Simmons 2006; Dee et al. 2011). Further, to assess the moisture budget components, we use six-hourly temporal resolution for total precipitation, specific humidity, omega and, u and v components of winds at levels varying from the surface to troposphere.

Based on the vertically-integrated water budget at daily time-scale, the precipitation can be disintegrated as:

1 $$P= E-\left[\omega .\frac{\partial q}{\partial p}\right]- \left[V.\nabla q\right]- \left[\frac{\partial q}{\partial t}\right]$$

where, E (mm/day) is surface evaporation into the atmosphere, $\omega$ (Pa/day) is the vertical velocity, $V$(m/day) is the horizontal wind, q (kg/kg) is the specific humidity and p (Pa) is the atmospheric pressure. Brackets denote a mass-weighted vertical integral from the surface (ps) to the top of the atmosphere (p = 0). It is defined for a quantity A, as [A] = ${\int }_{0}^{{p}_{s}}A\frac{dp}{g}$, where g is the acceleration due to gravity (m/day^2^). This equation is rather well established in the scientific community, and therefore we have not provided the complete derivation of the moisture budget here. Interested readers can refer to Trenberth and Guillemot (1995), Trenberth (1997), Trenberth and Guillemot (1998) and Trenberth et al. (2007) for more details. Equation 1 can then be expressed as:

2 $$P = { }E + { }V_{adv} + H_{adv} - { }\partial_{t} q.$$

where, ${V}_{adv}, {H}_{adv}$ and ${\partial }_{t}q$ represent the vertical moisture advection, the horizontal moisture advection and the time derivative of q, respectively. All the respective variables are taken from the JRA and ERA- interim reanalysis product.

Further, ${\partial }_{t}q$ corresponds to the change in the atmospheric moisture storage. The contribution of this term to the moisture budget is usually negligible compared to the other terms in Eq. 2, when the timescale is longer (for example, monthly timescale), and can be neglected. Considering this, the change in the longer timescale—mean precipitation can therefore be represented as (Oueslati et al. 2019):

3 $$\Delta P= \Delta E+ \Delta {V}_{adv}+ \Delta {H}_{adv}$$

Numerous studies have attempted to quantify the individual contributions of dynamic and thermodynamic components to precipitation extremes (e.g. Schaller et al. 2016; Vautard et al. 2016). However, these studies have considered mean sea-level pressure (SLP) as a proxy for a dynamic component. The mean SLP pattern can only describe the low-level horizontal atmospheric circulation, neglecting the influences of vertical motion, which controls the initiation and strength of convection (Oueslati et al. 2019). On the other hand, studies such as O’Gorman and Schneider (2009b), Vittal et al. (2016) and Gusain et al. (2019) have demonstrated the considerable role played by the vertical motion in the space–time variation of precipitation extremes. In view of this, the present study explicitly considers the vertical moisture advection and further decomposes it to dynamic (*Dyn*) and thermodynamic (*Thermo*) components following the framework provided by Oueslati et al. (2019). To necessitate this, the vertical advection component (V_adv_) of Eq. 1 and the *Dyn* and *Thermo* can be extracted as;

4 $$\Delta {V}_{adv}= Dyn+ Thermo= -\left[\Delta \omega .\frac{\stackrel{-}{\partial q}}{\partial p}\right]- \left[\stackrel{-}{\omega } . \Delta \frac{\partial q}{\partial p}\right]$$

The *Dyn* in Eq. 4 relates to the changes in vertical velocity during the extreme event. In contrast, the *Thermo* majorly relates to atmospheric water vapor changes, which broadly follows the concept of the CC equation. The overbar in Eq. 4 indicates the 1958–2018 (1981–2018) climatological mean for the JRA-55 (ERA-Interim) reanalysis product.

We first demonstrate the individual contributions of *Dyn* and *Thermo* to the two recent extreme precipitation events, which happened over the Kerala (2018) and Uttarakhand (2013) regions of India. The IMD reported that from early June to August of 2018, the amount of rainfall over Kerala was 40% higher than during normal years (Sudheer et al., 2019). This substantial and abnormal rainfall over the region produced a huge flood, which resulted in a tremendous impact on the socio-economy. It has been reported that over 483 people lost their lives, and over 1.4 million people were displaced from their homes (Muttarak and Dimitrova 2019). Similar to the Kerala event, the Uttarakhand event is also considered to be one of the most devastating events of recent times. This event initiated on 13th June 2013, with more than 340 mm of rainfall which is almost 375% more than the climatic normal during JJAS (Dube et al. 2014). This caused floods in the region and consequently had a detrimental effect on life and property. We further extend our analysis by considering several other recent events that happened over India from 2005 to 2018. Here, we have identified 26 extreme precipitation events in India from this period (Table S1) based on the annual reports of the India Meteorological Department (https://metnet.imd.gov.in/phps/publications_imdarep.php) and other relevant articles (Tomar 2012; Sherly et al. 2015) over the different parts of the nation. Table S1 shows the 26 extreme precipitation events over India; however, it should be noted that some of the incidents happened outside of monsoon months (JJAS) and the events set also contains events for regions which are not in the core monsoon zone (CMZ) [18.0°N–25.0°N, 65.0°E–88.0°E]. Considering this, we estimate the individual contributions from *Dyn* and *Thermo* for each event.

Further, to understand the processes represented by climate models, we have selected the latest suite of CMIP6 models (Eyring et al. 2016). The historical simulations from six different low and medium-resolution GCMs (Table S2) of CMIP6 were selected based on the availability of data (precipitation, specific humidity, and vertical velocity) in the Earth System Grid Federation (ESGF) (in May 2019). A recent study by Gusain et al. (2020) showed that these CMIP6 simulations, even though a small subset, are able to capture the characteristics of summer monsoon rainfall better than CMIP5 for both mean and rainfall extremes. The summer monsoon is characterized by heavy rainfall over the windward side of the Western Ghats as well as at the foothills of the Himalayas in Northeast (NE) India due to the orographic effect of the mountain range. These features are very well captured in CMIP6, better than in CMIP5. The bias against observations is also comparatively small in CMIP6 and a similar range of benefits has been observed in simulated summer precipitation extremes as well. For these reasons, we proceed with our analysis considering the CMIP6 model simulations. The model outputs are interpolated to the coarsest resolution among the models considered. To identify the extreme precipitation events, we used the peak-over-threshold (PoT) approach with a threshold of the 95^th^ percentile (Vittal et al. 2013) from 1950 to 2014 considering all days in the JJAS monsoon season. For these extreme events, we then computed the dynamic and thermodynamic contributions for each model over India [5.0°N–40.0°N, 60°E–100.0°E].

## Results and discussion

**Fig. 1:**
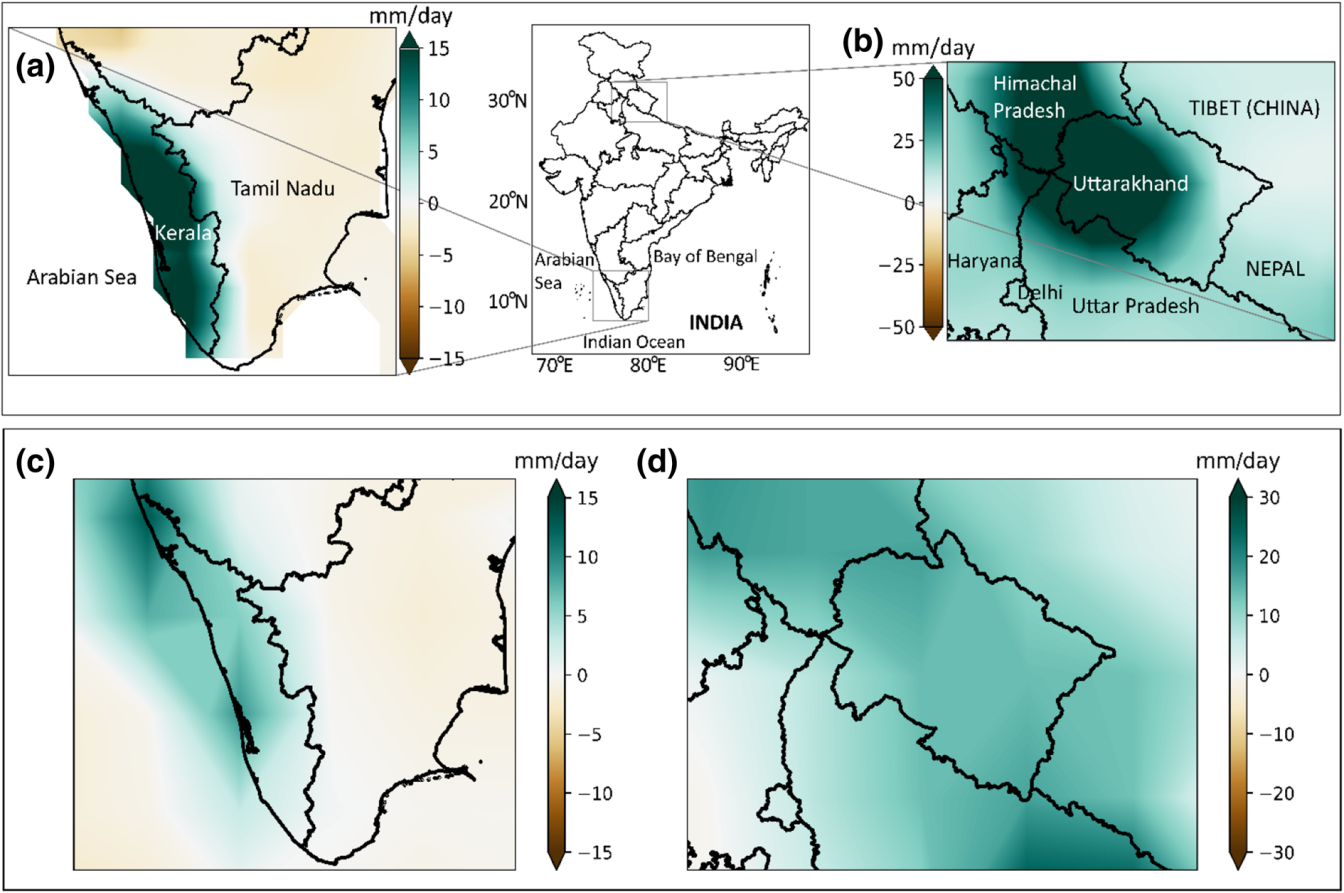
Precipitation anomalies for the extreme events. **a** Anomalies for Kerala (July 15 to Aug 18, 2018) and **b** Uttarakhand (June 13 to Jun 18, 2013) regions of India using CPC observations. These regions are indicated by the rectangular boxes shown in the India map, wherein the southern box represents Kerala and the northern box for Uttarakhand. (**c**, **d**) Similar to (**a**, **b**), but from the JRA reanalysis product. The anomalies are relative to the climatology from 1979 to 2018 for (**a**, **b**) and from 1958 to 2018 for (**c**, **d**), respectively

Initially, we focus our analysis on two extreme rainfall events that happened over India, in Kerala, and Uttarakhand during 2018 and 2013, respectively. The impacts caused by these two events could have been undeniably reduced with accurate forecasts and preparation. In the case of the Kerala event, the forecast from the IMD suggested maximum probability for normal monsoon rainfall and low probability for deficient rainfall during the monsoon season of 2018, which under-estimated the extreme event (Govt. of Kerala 2018). This incapability in forecasting extreme events implies an improper understanding of the mechanisms responsible for extreme events over India. With this objective, we proceed with our analysis to understand the mechanism of precipitation extremes during the two events. However, before proceeding, we estimate the precipitation condition from the observed CPC dataset for the two regions. For this purpose, we analyze the precipitation anomaly for these two regions during the respective period of extreme events. Figure 1a shows the precipitation anomaly for Kerala during the period 15 July to 18 August, 2018 is estimated relative to the summer monsoon rainfall climatology spanning from June–September from 1979 to 2018. As with the previous reports of IMD and other literature, here we also notice a high positive precipitation anomaly, confirming that this is an extreme event. We see similar results for the Uttarakhand region (Fig. 1b) for the 5-day period from June 13 to Jun 18, 2013 when the extreme precipitation event occurred.

As we use the reanalysis datasets to understand the large-scale dynamics related to these two extreme events, it is essential to evaluate the precipitation extremes produced by this reanalysis product. Figures 1c, d confirm the capability of the JRA-55 reanalysis product in reproducing the extreme precipitation characteristics for both the Kerala and Uttarakhand regions. ERA-Interim can also represent these extreme events (Figure S1); however, the anomalies were estimated relative to the climatology from 1979–2018, shorter duration compared to that of JRA-55 (1958 to 2018). Regardless of the data type and duration, it is evident from this analysis that the extreme precipitation events are captured by all the considered observed and reanalysis data products.

**Fig. 2:**
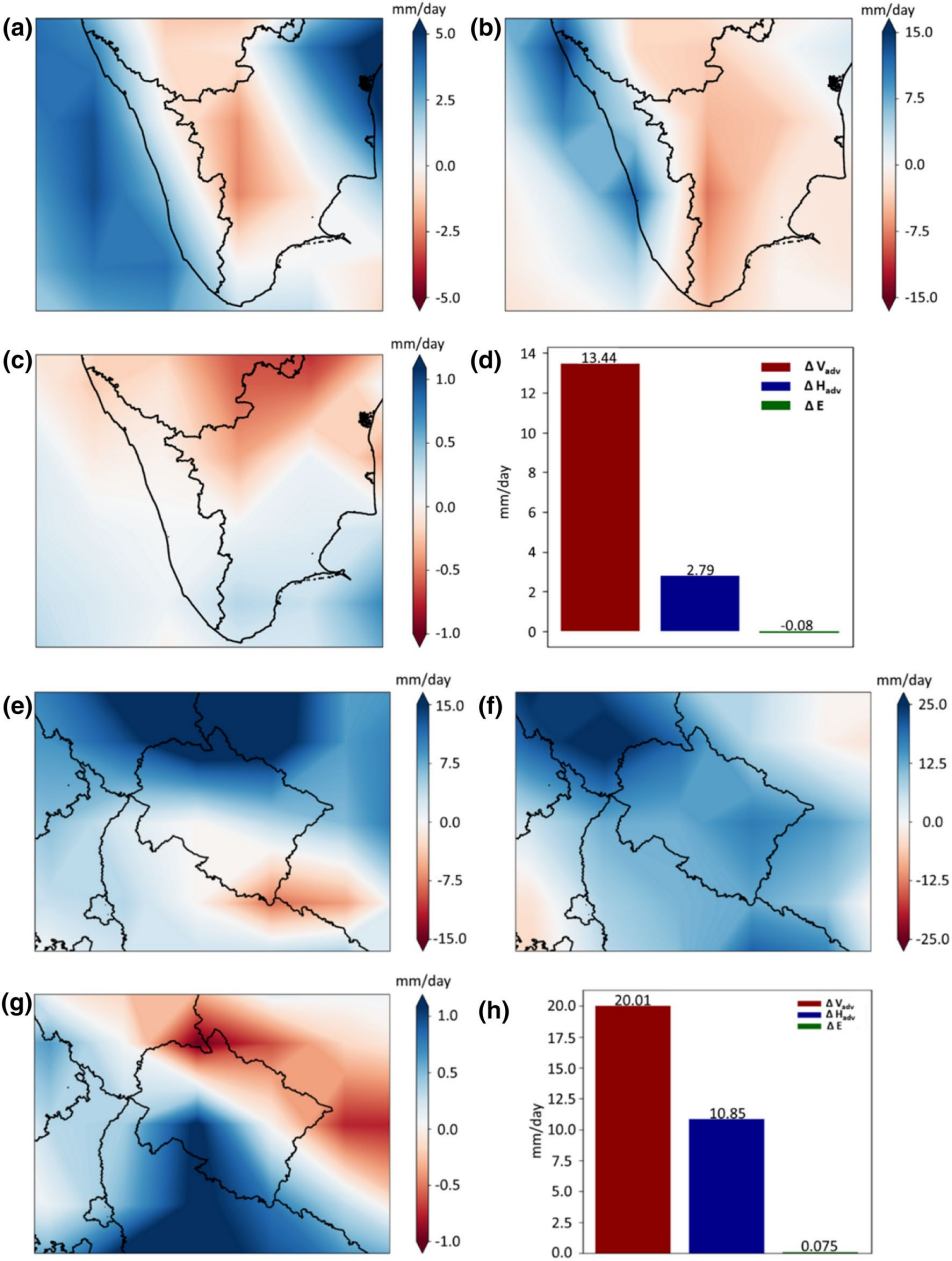
Anomalies for the water-budget components during the extreme event for Kerala and Uttarakhand. (**a**) Horizontal moisture advection, (**b**) Vertical moisture advection, (**c**) Surface evaporation and (**d**) the three water-budget component contributions spatially aggregated over Kerala as indicated by the southern rectangular box in Fig. 1a, computed from JRA reanalysis product. The anomalies are relative to the climatology from 1958 to 2018. (**e**–**h**) are same as (**a**–**d**), but for Uttarakhand region extreme event

Next, we analyze the components of moisture budget, based on Eq. 3. Here, we show the changes in horizontal and vertical moisture advection and also the evaporation components during the event. Figure 2a shows the condition of horizontal moisture advection during the extreme event over Kerala from the JRA-55 reanalysis dataset, and we note a positive anomaly. Similarly, to horizontal advection, the anomalies of vertical advection (Fig. 2b) are also positive; however, the magnitude is much larger than that for horizontal moisture advection. In the case of the evaporation component of the moisture budget, the changes are negligible (Fig. 2c). The overall contributions of these individual components for the spatially aggregated rainfall over Kerala for the extreme event suggests a stronger response from the vertical moisture advection component than that of horizontal moisture advection and evaporation (Fig. 2d). A similar split is obtained for the Uttarakhand extreme event (Fig. 2e–h), with vertical moisture advection dominating the other components of the moisture budget.

These positive anomalies of vertical moisture advection prompted the moistening of the troposphere mainly by the vertical transport of moisture, simultaneously sustaining the low-level moisture convergence. On the other hand, we note relatively small changes in the horizontal moisture advection during the events, although the changes are positive for both. This means that drying of the troposphere through the transport of dry air to the rainy regions is further minimized, which could also help to increase the precipitation intensity (Zhu and Hendon 2015). Nonetheless, from our analysis, it is clear that the positive anomaly of vertical advection is the main driver for the increased precipitation. Thus, the combination of abundant availability of moisture in the atmospheric column and increased vertical motion of the atmosphere are the main cause of the extreme precipitation events over both the Kerala and Uttarakhand regions.

**Fig. 3:**
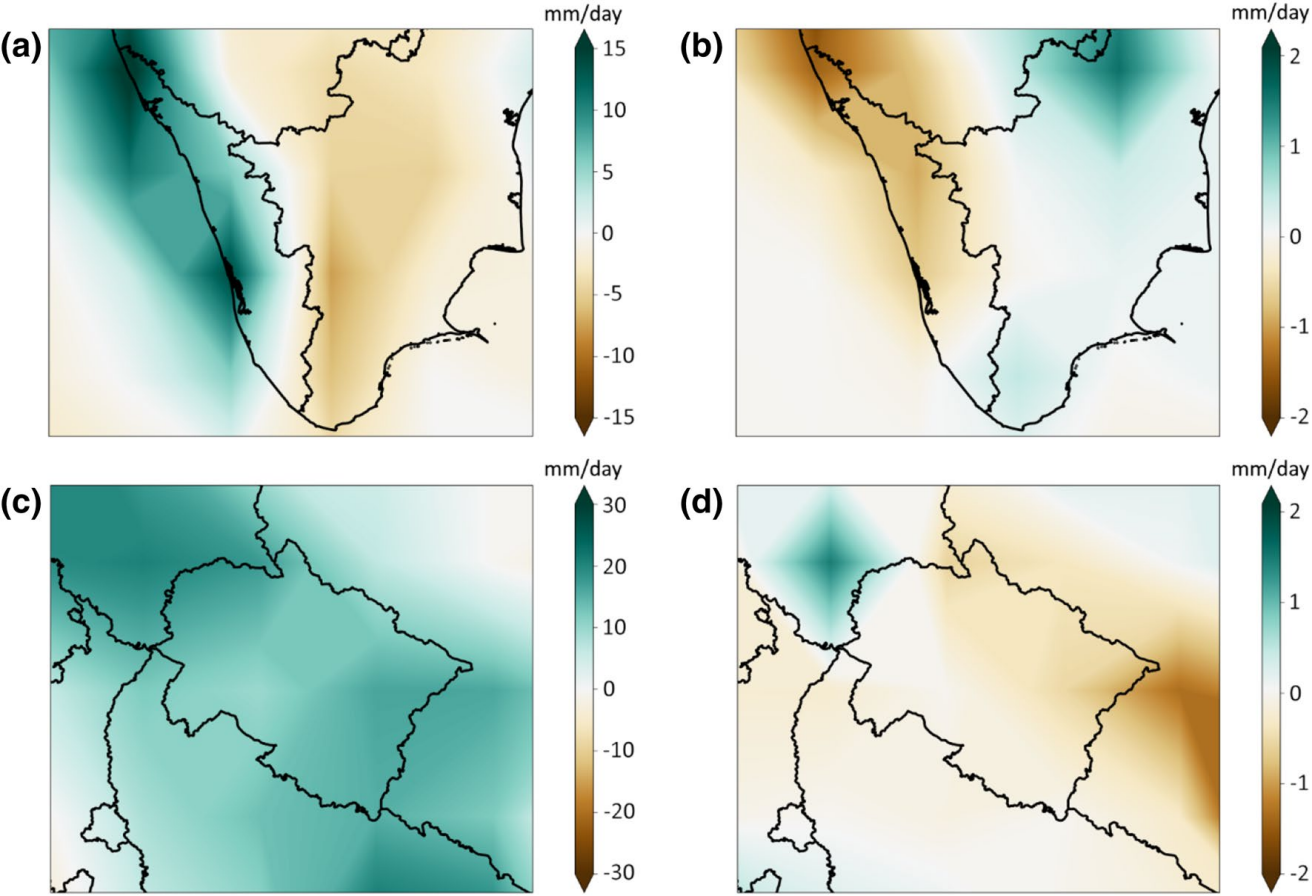
Anomalies of dynamic and thermodynamic components. (**a**) Dynamic component and (**b**) thermodynamic component anomalies for Kerala region extreme event. (**c**, **d**) are similar to (**a**, **b**), but for Uttarakhand region extreme event. The anomalies are computed from the JRA reanalysis product, which is relative to the climatology from 1958 to 2018

These results perhaps indicate the importance of considering the vertical motion of the atmosphere. To further understand the mechanisms causing extreme precipitation events over these regions, we now focus on the predominant driver, i.e., vertical moisture advection. Based on Eq. 4, we divide the vertical moisture advection into *Dyn* and *Thermo*. These contributions compute the vertically-integrated dynamic and thermodynamic changes and also include the influence of temperature lapse-rate changes (Kröner et al. 2017). Figure 3 illustrates using JRA-55 that *Dyn* is the main contributor to the vertical advection of moisture over the two considered regions. We obtain similar results for ERA-Interim (Figure S2). This further confirms our result that *Dyn* plays a more significant role than *Thermo* in inducing the extreme precipitation in the analysed Kerala and Uttarakhand events.

**Fig. 4:**
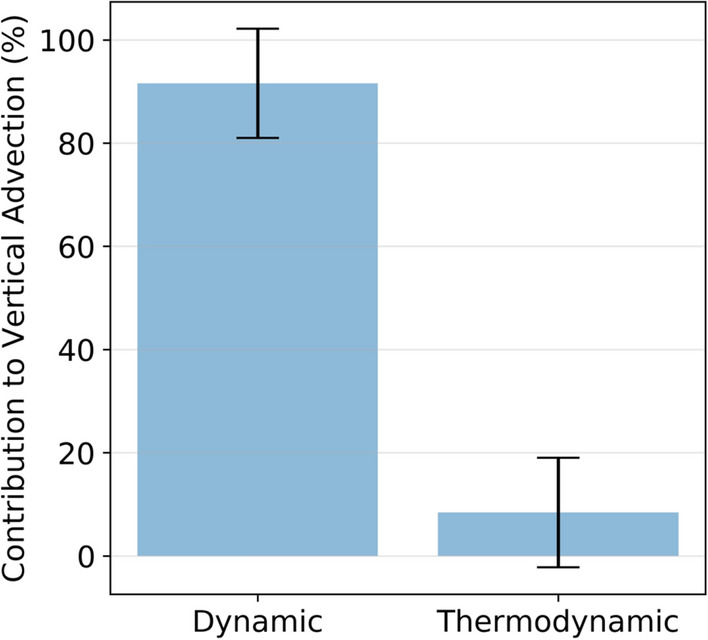
Comparison of contributions of dynamic and thermodynamic processes to the vertical advection component of the moisture-budget. The contributions are computed considering spatial aggregation over the different parts of India where the extreme events occurred. Refer to Supplementary Table S1 for detailed information. The gray bars represent the 90% confidence limits based on the sampling distribution of the mean of all the considered events

The two events discussed above are recent extreme precipitation events over India. However, to validate our results, we now consider other extreme events that have occurred over different regions of India. From reports available from IMD and other studies (Tomar 2012; Sherly et al. 2015), we consider 26 extreme precipitation events that have occurred over the last two decades (Table S1). For this purpose, *Dyn* and *Thermo* are estimated by spatially averaging the proxies of vertical moisture advection, viz., vertical velocity and specific humidity, during the extreme precipitation event. Figure 4 shows the overall contributions from these for the 26 extreme events and indicates that *Dyn* is the main contributor to the vertical moisture transport, which contributes more than 90% (mean for the considered extreme events), as already observed for the two case studies. We note the range of *Dyn* for these events is consistently high (represented by the uncertainty bar in Fig. 4). On the other hand, the contribution of *Thermo* is low for all events. This analysis further cements our result that *Dyn* contributions are larger than *Thermo* for extreme precipitation events over India. Our results are in the alignment with the recent study performed by Ali and Mishra (2018) who performed an analysis to understand the dynamic and thermodynamic contributions to precipitation extremes, but rather focusing only on urban locations in India. The precipitation extreme scaling used in their study were derived using an *energy budget* rather than a *water/moisture budget*, which allows the definition of a thermodynamic component, with no dependence on relative humidity. Also, because of the weak horizontal temperature gradient, especially in the Tropics, it eliminates the need for horizontal advective terms. A detailed description of the precipitation extreme scaling can be found in O'Gorman and Schneider (2009a, b), Muller et al. (2011) and Pfahl et al. (2017). On the other hand, our study performed a comprehensive analysis of the dynamic and thermodynamic contributions to precipitation extremes for the whole India, not limited to urban locations. Moreover, the motivation of the present study is to examine the individual contribution of water budget components to extreme events, including both evaporation and horizontal advection. Therefore, we preferred to use the equation derived from the moisture budget (Eqs. 1–3) than for the energy budget.

**Fig. 5:**
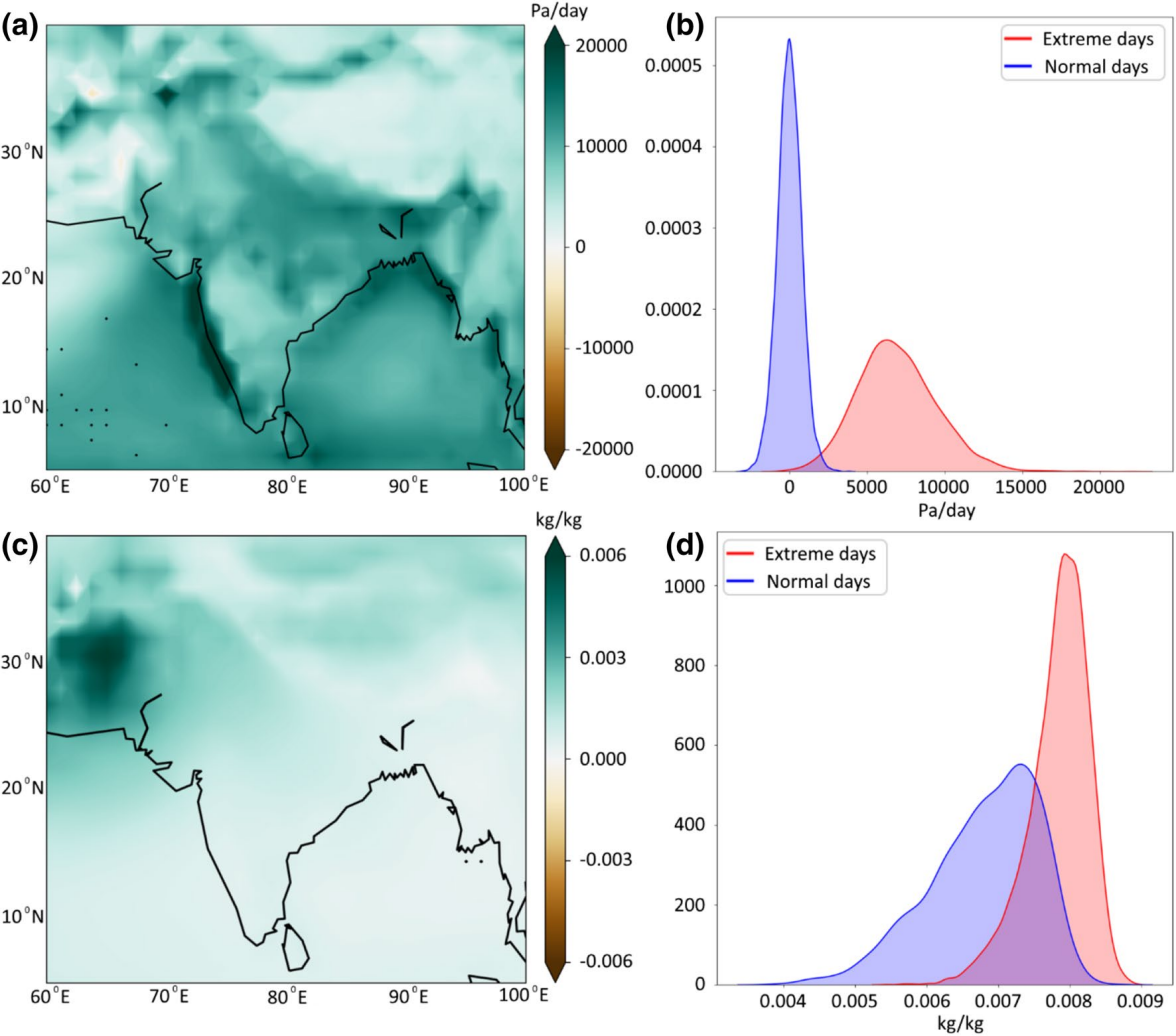
Changes in (**a**) vertical velocity and (**c**) specific humidity computed during normal and extreme monsoon days. Comparison of spatially aggregated (**b**) vertical velocity and (**d**) specific humidity between normal (blue) and extreme days (red) over the core monsoon zone of India [18.5°N–25.5°N, 74.5°E–84.5°E] in terms of probability distribution function (PDF)

Equation 4 depicts that changes in the vertical velocity (omega) are the main contributor to the *Dyn* contribution. In contrast, the changes in specific humidity are more related to the *Thermo* contribution. Therefore, we now assess the changes in vertical velocity and also specific humidity during extreme events. In this case, the precipitation extreme is defined based on PoT, considering the 95th percentile as threshold. We examine the vertical velocity and specific humidity on days when the precipitation extremes exceed this threshold and compute the differences from ‘normal’ (the other) JJAS monsoon days spanning from 1958–2018 from the JRA-55 reanalysis product. Figure 5a shows the increase in vertical velocity during the extreme precipitation days. The increases are more prominent in the dominant monsoon regions such as the western Ghats and North central parts, which encompasses the core monsoon zone (CMZ) of India. The average rainfall over the CMZ is used to define the active and break events and also used to determine the strength of the monsoon (Rajeevan et al. 2006). Thus, CMZ is considered the critical area for Indian monsoons. Figure 5b shows the vertical velocity during normal and extreme days for the CMZ region in terms of its probability density function (PDF). It is clear from this analysis that the vertical velocity during extreme precipitation days is significantly higher than that for the normal monsoon days. We also notice a positive change in specific humidity (Fig. 5c, d); however, these changes are not so prominent and significant as compared to vertical velocity.

**Fig. 6:**
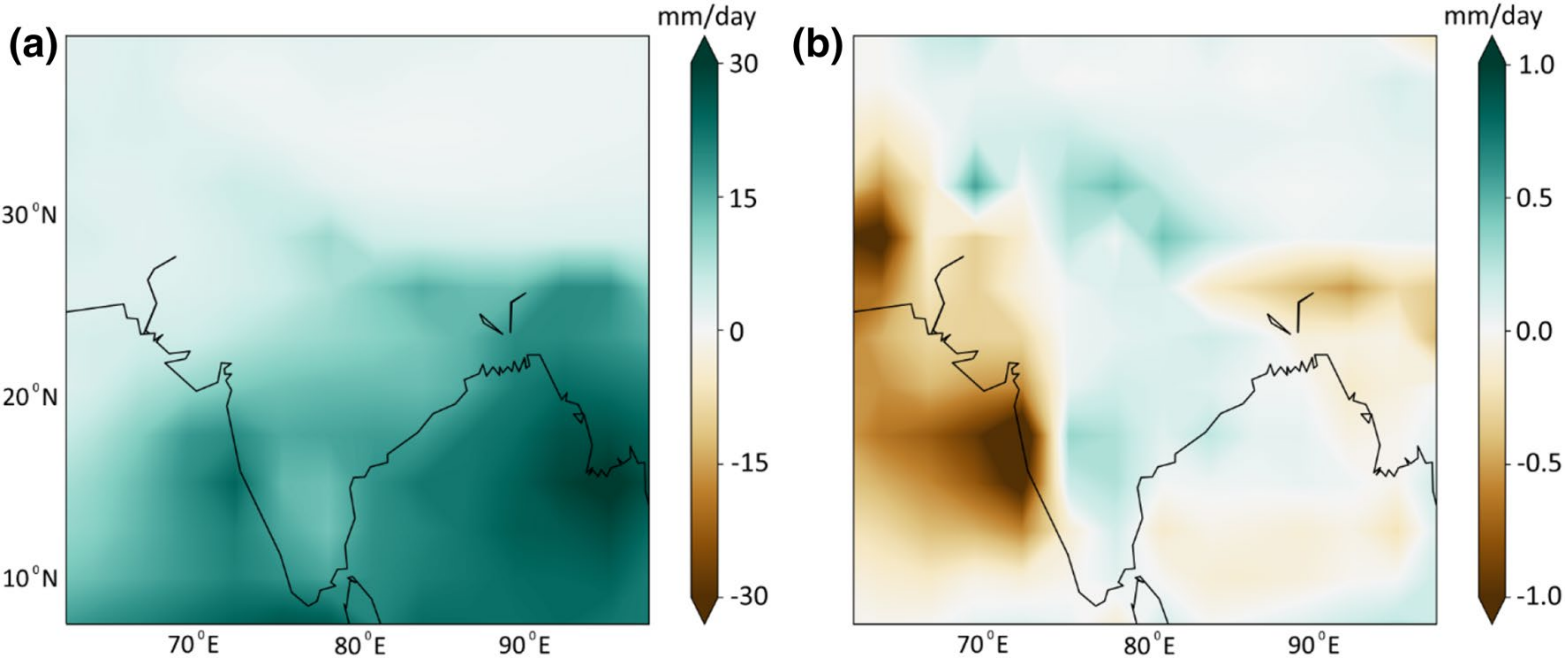
Evaluating the contributions from the CMIP 6 GCMs. Anomaly of (**a**) dynamic, and (**b**) thermodynamic component during the extreme events from the GCMs. For representation, we have considered multi-model mean from six individual GCMs. The details of these models are provided in supplementary Table S2. Refer to Supplementary Fig. S3 and S4 for individual model contributions of dynamic and thermodynamic components, respectively

It is now clear from the analysis with observed data that the *Dyn* plays an essential and crucial role in causing extreme precipitation events over India. However, the majority of climate impact assessment is based on simulations from climate models. Previous studies have reported on the inability of climate model simulations to reproduce precipitation extremes, especially in the tropical region (O’Gorman and Schneider 2009a, b). Further, Saha et al. (2014) and Sabeerali et al. (2015) reported the failure of climate models to capture recent trends in the ISMR. However, these studies used CMIP5 climate model simulations which failed to capture feedbacks from local scale features (such as topography, land-use changes, etc.) in reproducing present climatic conditions (Ghosh et al. 2016; Jain et al. 2019). To overcome these limitations, improved climate model simulations under the sixth phase of CMIP have been released recently. We now use a selection of these CMIP6 climate models to evaluate the response of *Dyn* and *Thermo* components during extreme events. As with the observations, the *Dyn* surpass the contribution of *Thermo* to vertical moisture advection (Fig. 6). A recent study by Gusain et al. (2020) showed that CMIP6 could represent the pattern of precipitation extremes over India better than CMIP5. We speculate from our analysis that the better representation of changes in *Dyn* is one of the main reasons for it, along with significant improvement in the deep convective schemes (Wu et al. 2019), cloud fractions (Vignesh et al. 2019) and improved horizontal and vertical resolutions (Herold et al. 2017).

## Conclusions

The prediction of rainfall over the extra-tropical region is relatively more straightforward than that of tropical areas. It is difficult to predict the ISMR, and more specifically its extreme aspects, because the monsoon is a unique tropical system that is strongly influenced by sub-seasonal variability (Goswami and Xavier 2005). Moreover, climate models have deficiencies in representing the response of precipitation extremes to global warming in tropical regions (O’Gorman and Schneider 2009b). Previous studies have tried to understand the mechanisms behind changes to precipitation extremes with warming and found results which contradict the classical theory of the CC equation (Emori and Brown 2005; Dairaku and Emori 2006; Pfahl et al. 2017); and suggests a considerable gap in ISMR's extreme predictability. It indicates that there is a need to understand the mechanisms for modulating the ISMR extreme to attain a good prediction/forecast skill, or for future projections.

Here, while evaluating the components of the moisture budget during extreme precipitation events, we show that vertical moisture advection plays a crucial role, compared to horizontal advection and evaporation components. However, we also found that though the contribution of horizontal moisture advection is small during the event, it provides a necessary precursor moisture condition. Based on the framework provided by Oueslati et al. (2019), we divided the vertical moisture advection into *Dyn* and *Thermo*. These two components are relevant matrices in this context, as they yield a proper physical mechanism at all vertical levels of the atmosphere, and these matrices have been used in extreme event attribution in many studies (e.g. Yiou et al. 2017). The results from our study show that atmospheric circulation is one of the crucial elements causing recent precipitation extremes events over India. Further, we comprehensively show that these events were majorly driven dynamically by inducing stronger vertical motions, which basically increases the moisture content of an atmospheric column and subsequently strengthens the convection. Overall, from the observed datasets, we demonstrate that the *Dyn* contribution surpasses the contribution from *Thermo* for all the extreme events examined for India.

As with the observations, the recent climate model simulations from CMIP6 also revealed the importance of *Dyn* in causing precipitation extremes over India. A recent study by Gusain et al. (2020) showed that the CMIP6 models simulate precipitation extremes better than the CMIP5 models. We speculate the more precise representation of *Dyn* during the extreme event is the leading cause for this. However, this needs to be further analyzed in future studies. Also, the next step would be to evaluate how the dynamic and thermodynamic contributions identified in the present study would change in scenario simulations; and evaluating the anthropogenic influence in altering the *Dyn* and *Thermo* contribution is an important and essential aspect in improving the future projections of extreme precipitation over India.

## Electronic supplementary material

Below is the link to the electronic supplementary material.

Supplementary file1 (DOCX 3191 kb)
