# Supplementary material for: Large-scale dynamics have greater role than thermodynamics in driving precipitation extremes over India
Source: Clim Dyn. 2020 Aug 3;55(9-10):2603–14. doi: 10.1007/s00382-020-05410-3 (PMC8550336; doi:10.1007/s00382-020-05410-3)
Supplement: Supplementary file 1 — Supplementary file1 (DOCX 3191 kb) [file 382_2020_5410_MOESM1_ESM.docx]

**Supplementary Information for**

**Large-scale dynamics have greater role than thermodynamics in driving precipitation extremes over India**

Naveen Sudharsan^1^, Subhankar Karmakar^1,2,3^, Hayley J Fowler^4^, Vittal Hari.^5*^

^1^Environmental Science and Engineering Department, Indian Institute of Technology Bombay, Mumbai-400076, India

^2^Interdisciplinary Program in Climate Studies, Indian Institute of Technology Bombay, Mumbai-400076, India

^3^Centre for Urban Science and Engineering, Indian Institute of Technology Bombay, Mumbai-400076, India

^4^School of Engineering, Newcastle University, Newcastle-upon-Tyne, UK

^5^Department of Computational Hydrosystems, Helmholtz Centre for Environmental Research – UFZ, Leipzig, Germany

Manuscript submitted to

***Climate Dynamics***

***Corresponding author:** Vittal Hari.; email:- vittal.hari@ufz.de

**
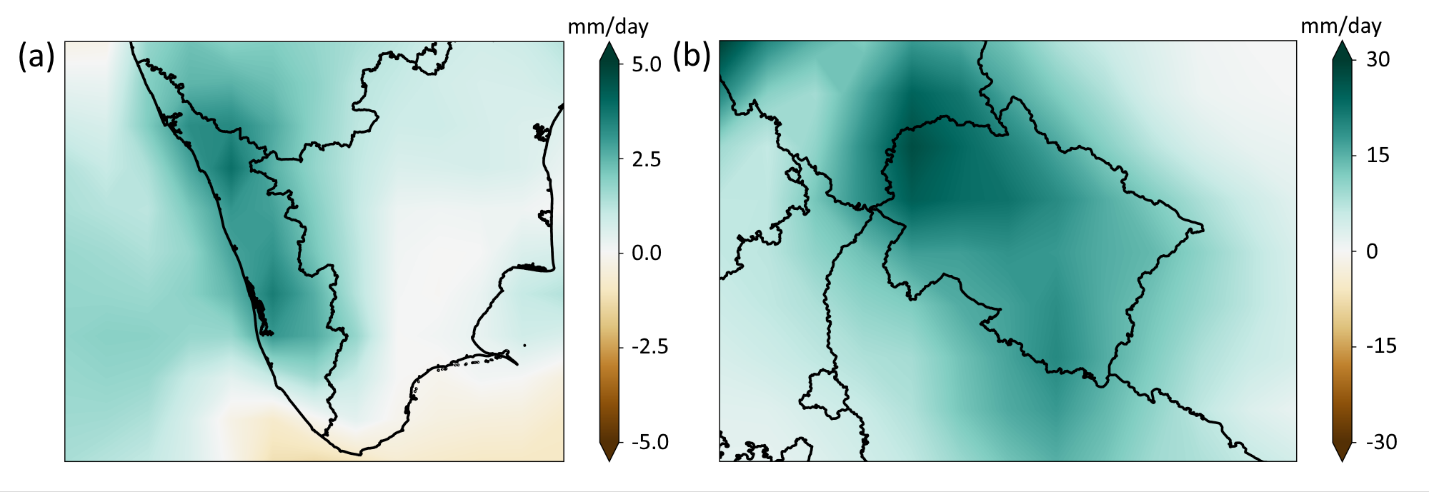
**

**Supplementary Figure S1:** Anomalies of precipitation during the extreme precipitation event for July 15 to Aug 18, 2018) and (b) Uttarakhand (June 13 to Jun 18, 2013) from the ERA-Interim reanalysis product. The anomalies are relative to the climatology from 1980-2018.

**
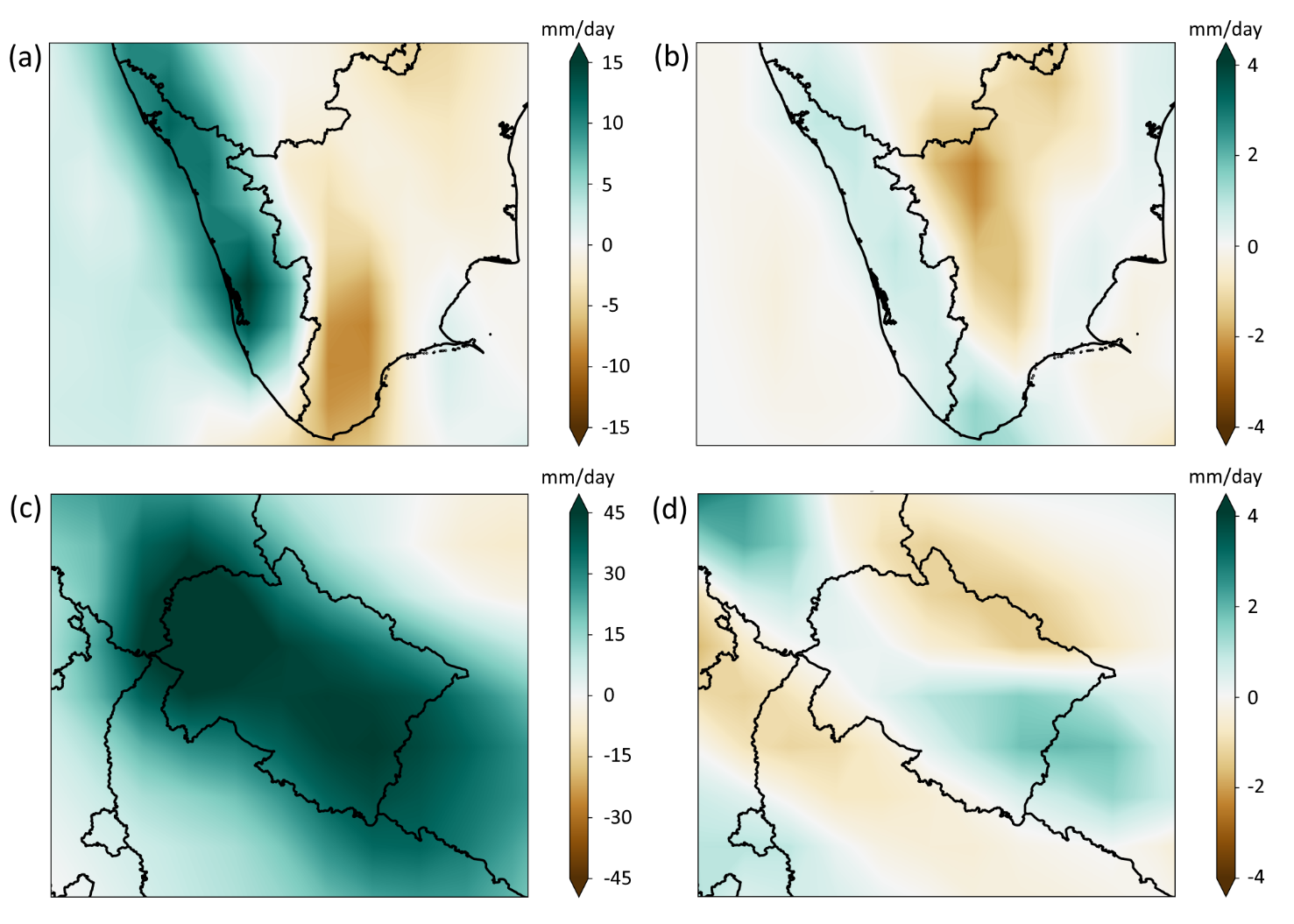
**

**Supplementary Figure S2: Anomalies of dynamic and thermodynamic components.** (a) Dynamic component and (b) thermodynamic component anomalies for Kerala region extreme precipitation event. (c) and (d) are similar to (a) and (b), but for Uttarakhand region extreme event. The anomalies are computed from the ERA-Interim reanalysis product, which is relative to the climatology from 1980 to 2018.

**
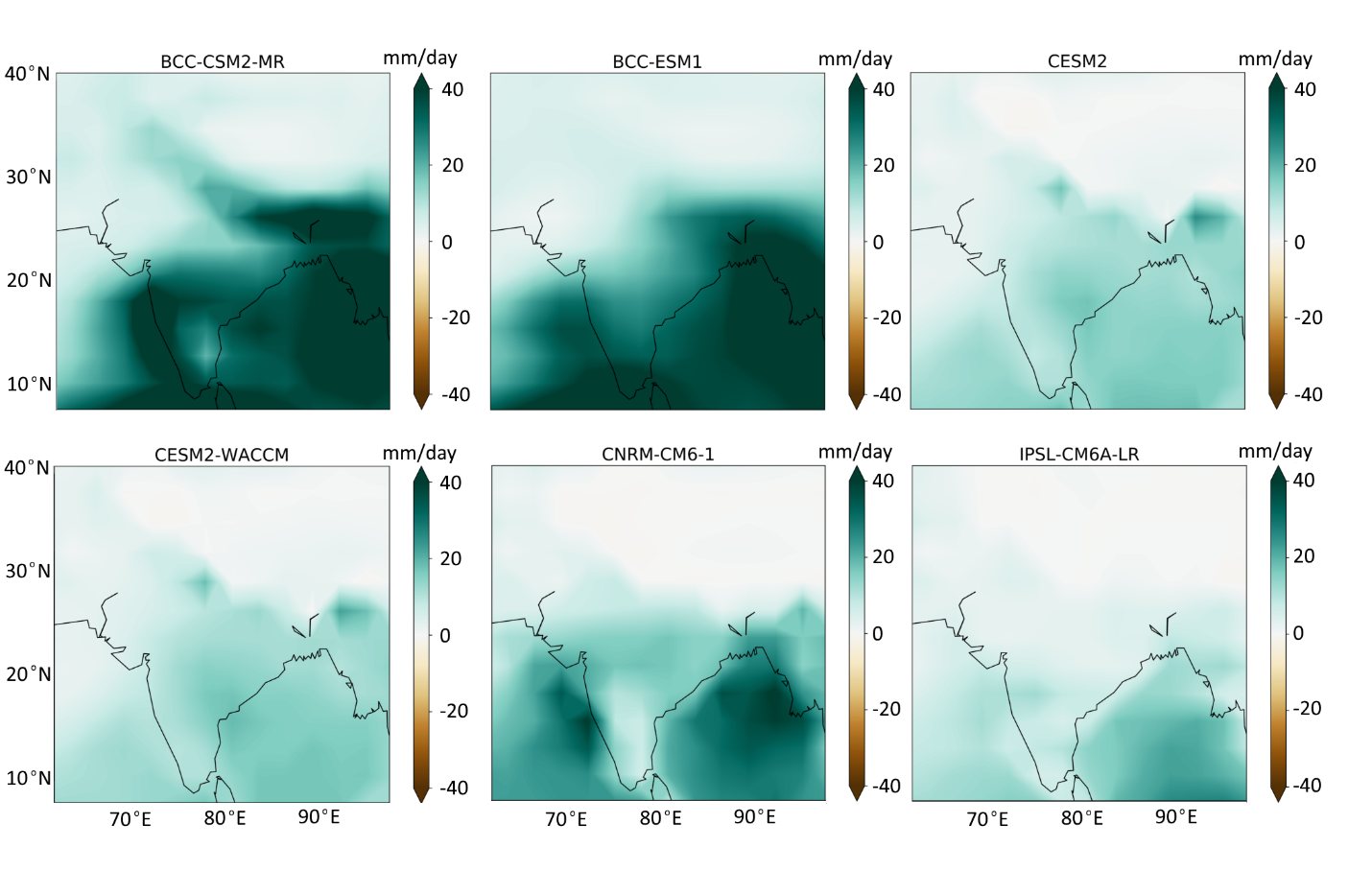
**

**Supplementary Figure S3:** Dynamic contributions as obtained from the individual GCMs.

**
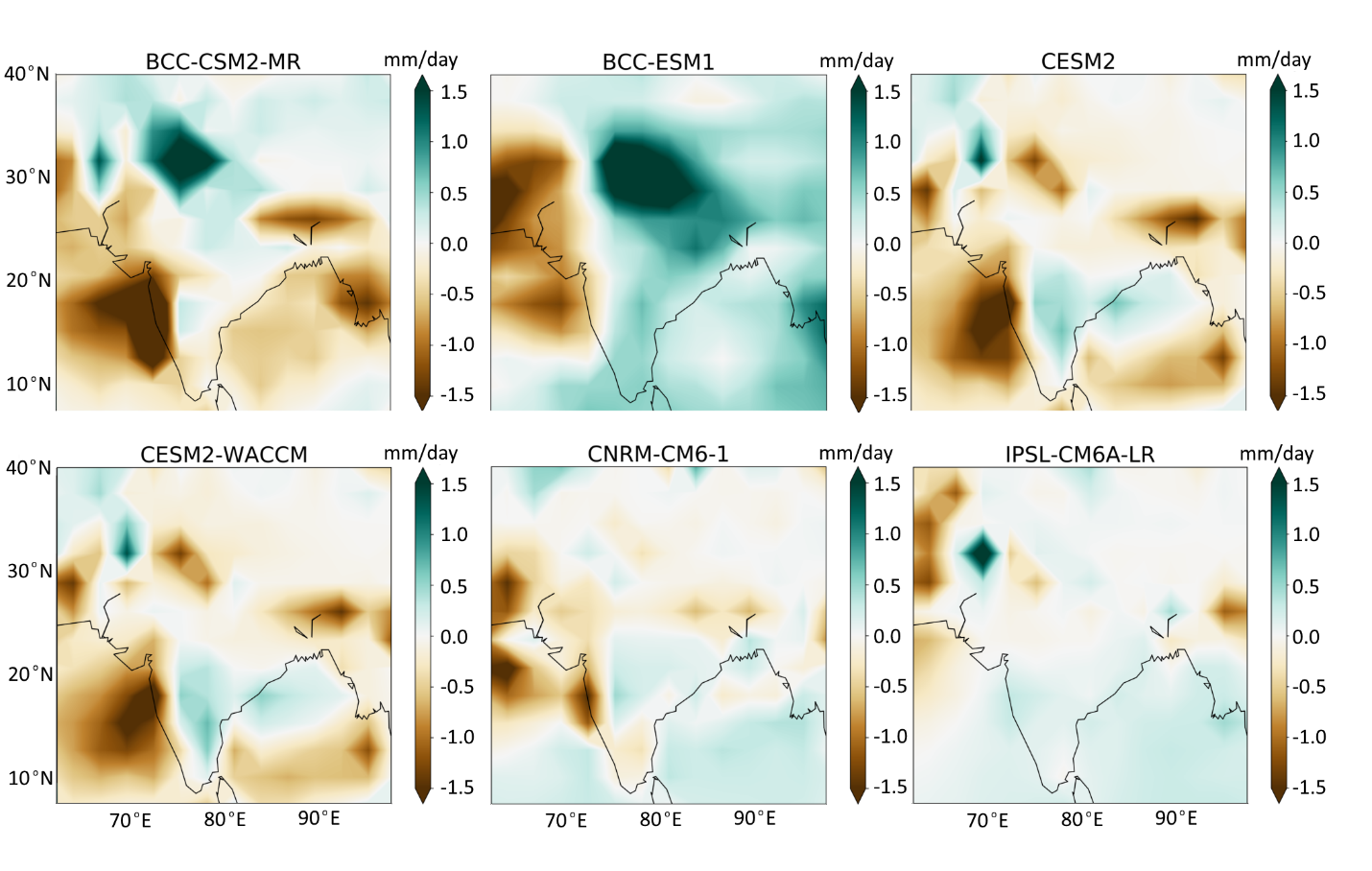
Supplementary Figure S4:** Thermodynamic contributions as obtained from the individual GCMs.

**Supplementary Table S1:** Details of extreme precipitation events considered over India in chronological order based on the information provided by IMD.

| **SI No.** | **Year** | **Month** | **Region** | **State** | **Total Precipitation (mm)** |
| --- | --- | --- | --- | --- | --- |
| 1 | 2005 | Jul | 15.6N to 22N, 72.6E to 80.8E | Maharashtra | 475 |
| 2 | 2005 | Jul | 33N to 37N, 72.4E to 80.3E | Jammu and Kashmir | 380 |
| 3 | 2008 | Jun | 21.6N to 27N, 85.76E to 89.89E | West Bengal | 370 |
| 4 | 2008 | Jun | 22N to 25.3N, 83.3E to 87.9E | Jharkhand | 400 |
| 5 | 2008 | Sept | 17.8N to 22.55N, 81.35E to 87.5E | Odisha | 350 |
| 6 | 2010 | May | 12.7N to 19.2N, 76.8E to 84.76E | Andhra Pradesh | 150 |
| 7 | 2010 | Nov | 8.1N to 13.6N, 76.3E to 80.3E | Tamil Nadu | 280 |
| 8 | 2010 | Nov | 12.7N to 19.2N, 76.8E to 84.76E | Andhra Pradesh | 130 |
| 9 | 2010 | Nov | 11.6N to 18.48N, 74E to 78.5E | Karnataka | 140 |
| 10 | 2011 | Sept | 24.3N to 27.5N, 83.35E to 88.1E | Bihar | 235 |
| 11 | 2011 | Aug | 21.1N to 26.8N, 74.1E to 82.73E | Madhya Pradesh | 365 |
| 12 | 2011 | Aug | 23N to 30.15N, 69.5E to 78.2E | Rajasthan | 165 |
| 13 | 2011 | Aug | 29.6N to 32.45N, 73.9E to 76.9E | Punjab | 400 |
| 14 | 2013 | Sept | 20.15N to 24.7N, 68.2E to 74.45E | Gujarat | 290 |
| 15 | 2013 | Jun | 29.6N to 31.45N, 77.7E to 80.97E | Uttarakhand | 500 |
| 16 | 2013 | Sept | 23N to 30.15N, 69.5E to 78.2E | Rajasthan | 390 |
| 17 | 2013 | Oct | 17.8N to 22.55N, 81.35E to 87.5E | Orissa | 340 |
| 18 | 2013 | Oct | 12.7N to 19.2N, 76.8E to 84.76E | Andhra Pradesh | 265 |
| 19 | 2015 | Jul | 20.15N to 24.7N, 68.2E to 74.45E | Gujarat | 290 |
| 20 | 2015 | Dec | 12.8N to 13.3N, 80E to 80.3E | Tamil Nadu | 500 |
| 21 | 2016 | Jul | 29.6N to 31.45N, 77.7E to 80.97E | Uttarakhand | 325 |
| 22 | 2016 | Jul -Aug | 21.1N to 26.8N, 74.1E to 82.73E | Madhya Pradesh | 945 |
| 23 | 2016 | Jul | 24.25N to 28N, 89.7E to 95.85E | Assam | 475 |
| 24 | 2016 | Jul - Sept | 20.15N to 24.7N, 68.2E to 74.45E | Gujarat | 400 |
| 25 | 2017 | Jul | 20.15N to 24.7N, 68.2E to 74.45E | Gujarat | 440 |
| 26 | 2018 | Jul -Aug | 8.3N to 12.8N, 74.9E to 77.25E | Kerala | 1470 |

**Supplementary Table S2:** Details of CMIP6 - GCMs used

| **SI No.** | **Model** | **Modeling Centre** | **Variant Label** | **Resolution (lon×lat)** |
| --- | --- | --- | --- | --- |
| 1 | BCC-CSM2-MR | Beijing Climate Centre, China Meteorological Administration | r1i1p1f1 | 1.125°×1.125° |
| 2 | BCC-ESM1 | Beijing Climate Centre, China Meteorological Administration | r1i1p1f1 | 2.8125°×2.8125° |
| 3 | CESM2 | National Centre for Atmospheric Research, United States of America | r1i1p1f1 | 1.25°×0.9375° |
| 4 | CESM2-WACCM | National Centre for Atmospheric Research, United States of America | r1i1p1f1 | 1.25°×0.9375° |
| 5 | CNRM-CM6-1 | Centre National de Recherches Météorologiques/Centre Européen de Recherche et Formation Avancées en Calcul Scientifique | r1i1p1f2 | 1.4°×1.4° |
| 6 | IPSL-CM6A-LR | Institut Pierre-Simon Laplace | r1i1p1f1 | 2.5°×1.259° |
